# Protective effect of anisodamine hydrobromide on lipopolysaccharide-induced acute kidney injury

**DOI:** 10.1042/BSR20201812

**Published:** 2020-06-30

**Authors:** Feng Wan, Xiaoqiang Du, Huan Liu, Xueling He, Ye Zeng

**Affiliations:** 1 Institute of Biomedical Engineering, West China School of Basic Medical Sciences and Forensic Medicine, Sichuan University, Chengdu 610041, China; 2 Chengdu No.1 Pharmaceutical Co., Ltd. Chengdu 610031, China; 3 Laboratory Animal Center, Sichuan University, Chengdu 610041, China

**Keywords:** metabolic analysis, oxidative stress, renal injury, sepsis

## Abstract

Anisodamine hydrobromide (AniHBr) is a Chinese medicine used to treat septic shock. However, whether AniHBr could ameliorate septic acute kidney injury and the underlying mechanism were not investigated. In the present study, 18 male Sprague-Dawley rats (200–250 g) were randomly divided into control, lipopolysaccharide (LPS) and LPS+AniHBr groups. Rats were intravenously administrated with LPS or normal saline (for control). After 4 h, the rats were intravenously administrated with AniHBr (LPS+AniHBr) or normal saline at 4 h intervals. Hemodynamic parameters including blood pressure and heart rate were measured. The histopathologic evaluation of kidney tissues was performed. Lactate, creatine kinase, inflammatory cytokines and oxidative stress indicators were determined. Using Seahorse analysis, the metabolic analysis of mitochondrial stress and glycolytic stress in human renal proximal tubular epithelial cells treated with TNF-α in the presence of AniHBr was performed. AniHBr administration significantly reduced serum creatine kinase and lactate following LPS treatment. AniHBr significantly improved hemodynamics in sepsis rats including increase in the mean atrial pressure and reduction in the heart rate. AniHBr significantly attenuated LPS-induced TNF-α, IL-6 and IL-1β in serum, and LPS-induced TNF-α and IL-1β in renal tissues. The LPS-reduced SOD activity and LPS-increased MDA content were reversed by AniHBr. *In vitro*, TNF-α increased mitochondrial oxygen consumption and glycolysis, but inhibited the ATP generation, which was reversed by AniHBr. Thus, AniHBr protects against the LPS-induced inflammatory cytokines, mitochondrial dysfunction and oxidative stress, and thus attenuates the LPS-induced acute kidney injury, showing AniHBr is a promising therapeutic drug for septic kidney injury.

## Introduction

Sepsis or septic shock is the major cause of death from infection. Depending of the extent of the inflammatory response, the patients may suffer multiple organ failure, including the heart, liver, lungs and acute kidney injury (AKI). Sepsis is the greatest risk for septic AKI among hospitalized patients, which was account for approximately 30–70% of AKI patients. The incidence of sepsis is 29% in overall intensive care units and the in-hospital mortality rate is approximately 33% [1]. Therefore, it is important to develop diagnostic and treatment strategies to protect the patients from sepsis. In fact, there is still no effective drug available in the clinical setting.

Sepsis-induced AKI is closely modulated by inflammatory cytokines such as tumor necrosis factor α (TNF-α), which could induce most of septic symptoms and signs including AKI. It was demonstrated that suppression of TNF-α signaling by anti-TNF-α antibody could result in sepsis resistance [2]. The bacterial endotoxin lipopolysaccharide (LPS) is extremely strong stimulators of inflammatory reactions, which was administered to construct the sepsis animal model that is mostly studied. The LPS-induced AKI could be suppressed by inactivation of TNF-α signaling.

As adenosine triphosphate (ATP) generation was suppressed in septic patients, the failure of oxygen usage in mitochondria will damage the organic cell and tissues, as well as cause the metabolic abnormalities. High level of reactive oxygen species (ROS) is required for pathogen clearance and intracellular signaling. It was proposed the pathogenesis of septic-induced AKI based on an assumption that the adaptive responses to injury such as interplay among inflammation and dysfunctions of vascular microcirculatory and oxidative stress are driven by mitochondria [3].

Anisodamine hydrobromide (C_17_H_23_NO_4_·HBr, AniHBr) is an active ingredient extracted from the root of a Chinese specialty plant *Scopolia tangutica* maxim in 1956. The anisodamine has been synthesized and used clinically in China for improving the blood circulation in patients with organphosphorus poisoning or septic shock since 1956 [4] and was a proved powerful inhibitor of platelet aggregation possibly through inhibition of cyclo-oxygenase or thromboxane synthetase at 1982 [5]. It was implicated that anisodamine is an antioxidant and antagonist of muscarinic acetylcholine receptor to prevent free radical and thus protects against cellular damage [6]. Anisodamine could reduce the damage of myocardial mitochondria and thus inhibiting the cell apoptosis [7–9]. In recent, it was reported that anisodamine could significantly inhibit oxidative stress and attenuate the TNF-α in myocardial ischemia/reperfusion injured rats [10,11]. In mice with Shiga toxin-producing *Escherichia coli* infection, anisodamine administration suppressed *TNF-α* mRNA expression [12]. In glycerol-induced AKI, anisodamine administration reduced ROS-induced oxidative stress and thus protected against AKI [13]. Therefore, we hypothesized that the natural extract AniHBr prevent kidney from septic injury by suppressing the mitochondrial metabolism and subsequently mitochondrial dysfunction via suppression of oxidative stress and release of inflammatory cytokines.

In the present study, we aimed to investigate the role of AniHBr in renal injury in septic rats, and explore the effects of AniHBr on inflammation and inflammation-damaged mitochondrial function by *in vitro* experiments.

## Materials and methods

### Animals and experimental protocols

The present study is undertaken according to the recommendations in the Guide for the Care and Use of Laboratory Animals with approval by the Animal Research Ethics Committee of Laboratory Animals Center of Sichuan University (No.2019-5-15), Chengdu, China. Male Sprague-Dawley rats (200–250 g) were provided by the Experimental Animal Center of the Sichuan University (Chengdu, China). The rats were housed in solid-bottom cage at constant temperature (25 ± 2°C) and humidity (55 ± 10%) in a temperature-controlled facility in a standard breeding environment, with 12:12 h light/dark cycles in Laboratory Animals Center of Sichuan University. All rats were allowed access to food and water ad libitum, but they were fasted for 1 h before the experiments.

All rats were randomly divided into three groups as follows: control group (*n*=6), LPS group (*n*=6) and LPS+AniHBr group (*n*=6). The rats were kept grouped in cages (6 rats per cage). LPS from *Escherichia coli* O55:B5 (L2880; Sigma, Mo, U.S.A.) was administrated intravenously by tail vein injection with 5 mg/kg LPS in conscious rats. Rats were administrated intravenously by tail vein injection with AniHBr (the purity of C_17_H_23_NO_4_·HBr > 98.5%, Lot. 190501, Chengdu NO.1 Pharmaceutical Co., Ltd, China) with 3.6 mg/kg or an equal volume of vehicle (normal saline, for control group) at 4 h intervals. The first injection of AniHBr is at 4 h after LPS administration, and the last dose was given at 20 h after LPS administration. The mean arterial pressures and heart rates were measured by Medlab non-invasive pressure monitoring system (KEW, Nanjing, China). The blood specimens were collected from the orbital venous plexus at 24 h after LPS injection, and serum was isolated by centrifugation at 1500 ***g*** for 15 min at 4°C within 1 h after blood collection. After 24 h, all rats were euthanasia using carbon dioxide [14] and kidneys were isolated for further analysis. For histopathologic evaluation, part of each kidney was fixed in 4% paraformaldehyde solution, and then the remaining tissue was frozen immediately in liquid nitrogen and stored at −80°C.

### Histopathologic evaluation

The kidney tissues were dehydrated and embedded in paraffin, and then were sliced into 3 μm sections and stained with hematoxylin and eosin for histopathologic evaluation. Images were acquired with an Olympus DP70 microscopy with a digital camera (Olympus Optical Co, Ltd., Tokyo, Japan). Using ImageJ software v.1.52s (National Institutes of Health, U.S.A.), the swelling, vacuolar degeneration, necrosis and desquamation of tubular epithelial cells were graded using a semi-quantitative scoring method as previously described [13]: (I) <25% of cortical tubules; (II) 25–50% of cortical tubules; (III) 50–75% of cortical tubules; (IV) >75% of cortical tubules. The extent of LPS-induced renal injury and recovery by administration of AniHBr was performed. All histological evaluation was performed in a double blinded manner.

### Assessment of renal function, inflammation and oxidative stress

For renal function assessment, lactate (BC2235, Solarbio, Beijing, China) and creatine kinase (BC1145, Solarbio, Beijing, China) were measured using assay kits according to the manufacturer’s instructions. For inflammatory cytokines, serum TNF-α (Neobioscience, Shenzhen, China), IL-1β (Solarbio, Beijing, China) and IL-6 (Neobioscience, Shenzhen, China) were determined using commercial enzyme-linked immunosorbent assay (ELISA) kits. For ELISA, data were detected by absorbance at 450 nm using a Labserv K3 Touch microplate reader (ThermoFisher, U.S.A.).

Kidney tissues was gently homogenized in homogenization solution (10 mM Tris-HCl, 1 Mm EDTA, pH 7.4). After centrifuged at 3500 ***g*** for 10 min at 4°C, the supernatant was used for the measurement of malondialdehyde (MDA) concentration (A003-1, Nanjing Jiancheng Bioengineering Institute, Jiangsu, China) and superoxide dismutase (SOD) activity (A001-1, Nanjing Jiancheng Bioengineering Institute, Jiangsu, China) using assay kits according to the manufacturer’s instructions, and IL-6 level (Neobioscience, Shenzhen, China) was determined using the ELISA kit.

### *In vitro* cell culture, mito stress test and glycolytic stress test

Human renal proximal tubular epithelial cell line HK-2 (ATCC, Rockville, Md, U.S.A.) were cultured in Dulbecco Modified Eagle medium (DMEM, Hyclone, U.S.A.) supplemented with 10% fetal bovine serum (Tianhang, Zhejiang, China) and 1% penicillin/streptomycin (Invitrogen, ThermoFisher, U.S.A.) at 37°C with 5% CO_2_.

Cells were treated with or without a final concentration of 10 μg/ml AniHBr (Lot.190303, dissolved in ddH_2_O; Chengdu NO.1 Pharmaceutical Co., Ltd, China) in the presence of a final concentration of 10 ng/ml TNF-α (T6674, Sigma, U.S.A.) for 24 h. Then, the mito stress test was performed with the XF96 Extracellular Flux Analyzer (Seahorse Bioscience, MA, U.S.A.) according to the supper’s instructions [15]. After calibration (baseline), with an addition of 1 μM oligomycin, 2 μM carbonylcyanide p-(trifluoromethoxy) phenylhydrazone (FCCP) and 0.5 μM antimycin A, the oxygen consumption rate (OCR) was measured and then the basal OCR, maximal OCR and ATP-linked OCR (ATP production) were analyzed using Seahorse Wave software v.2.6.1 (Agilent, U.S.A.). With an addition of 10 mM glucose, 1 μM oligomycin and 0.5 mM 2-deoxyglucose (2-DG), the extracellular acidification rate (ECAR; glycolysis) was measured and analyzed. Graphs were performed using Garphpad Prism 7.0 software (Graphpad software, Inc., U.S.A.).

### Statistical analysis

Data are expressed as means ± SD from at least three independent experiments. All statistical analyses were performed using SPSS 25 (IBM, U.S.A.) with one-way analysis of variance (ANOVA) with least significant difference (LSD) post hoc test. Values of *P*<0.05 were considered statistical significant.

## Results

### AniHBr reduces serum creatine kinase and lactate concentrations in sepsis rats

**Figure 1:**
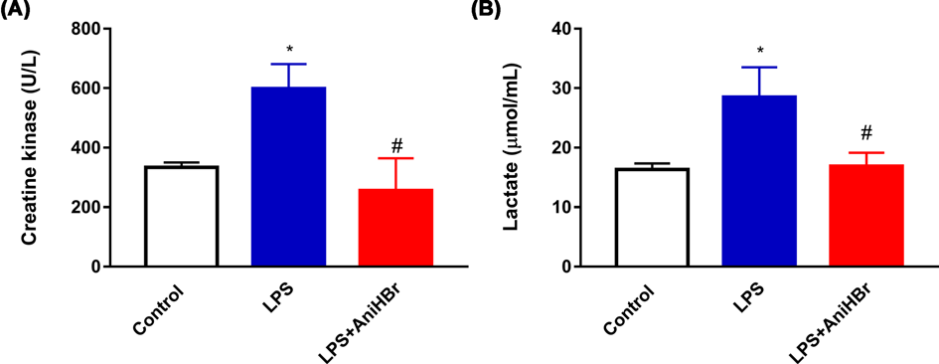
Serum creatine kinase (A) and lactate (B) concentrations at 24 h after the administration of LPS or vehicle (normal saline, control) The rats were treated with LPS, and then administrated with vechicle, or AniHBr. Mean ± SD, *P<0.05 vs. control; ^#^*P*<0.05 vs. LPS.

LPS rats showed higher serum creatine kinase compared with control rats after 24 h. Administration of AniHBr significantly suppressed the serum creatine kinase (Control group, 333.3 ± 17.2 U/l; LPS group, 598.0 ± 83.2 U/l; LPS+AniHBr, 255.7 ± 108.9 U/l) (Figure 1A).

Compared with control rats, LPS significantly increased the lactate concentrations at 24 h, but AniHBr administration significantly prevented this increase (Control group, 16.34 ± 1.0 μmol/ml; LPS group, 28.5 ± 5.0 μmol/ml; LPS+AniHBr, 16.9 ± 2.3 μmol/ml) (Figure 1B).

### AniHBr improve hemodynamics in sepsis rats

**Figure 2:**
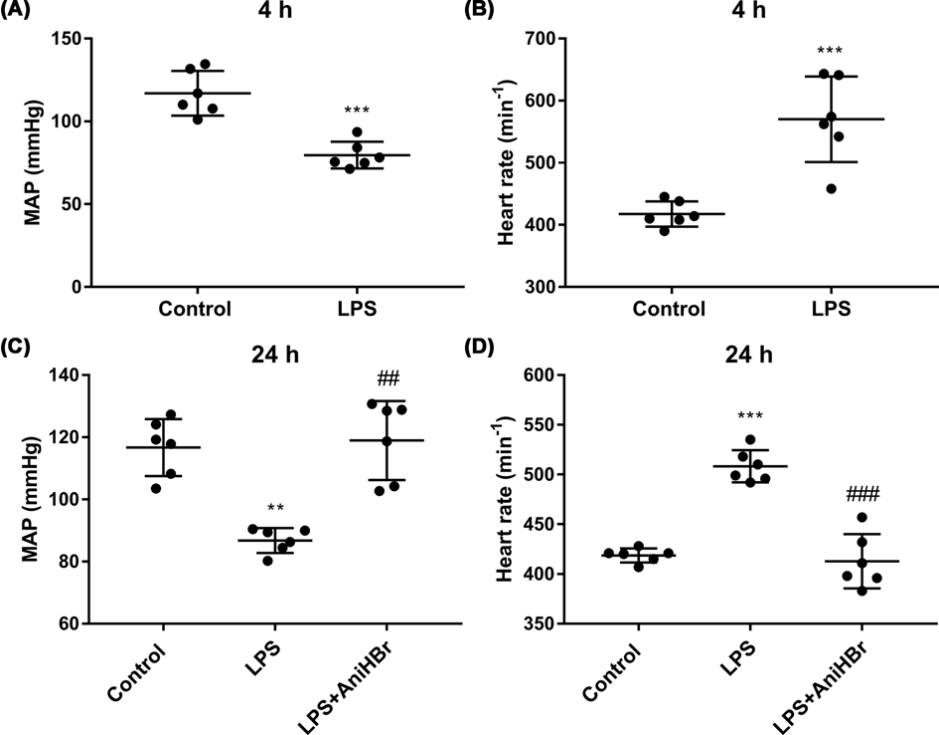
Hemodynamic parameters The mean atrial pressure (MAP; **A** and **C**) and heart rate (**B** and **D**) were measured at 4 h and 24 h after the administration of LPS or vehicle (normal saline, control). The rats were treated with LPS, and then administrated with vechicle, or AniHBr. Mean ± SD, **P<0.01, ***P<0.05 vs. control; ^##^*P*<0.01, ^###^*P*<0.001 vs. LPS.

The hemodynamic parameters including mean arterial pressure (Figure 2A,C) and heart rate (Figure 2B,D) were measured. LPS significantly increased the heart rate (Control group, 116.9 ± 13.5 s^−1^; LPS group, 79.6 ± 8.1 s^−1^; Figure 2B) and reduced the mean atrial pressure (Control group, 417.5 ± 20.5 mmHg; LPS group, 570.0 ± 69.0 mmHg; Figure 2A) at 4 h compared with control group. After 24 h, the heart rate of rats in LPS group was slightly reduced, compared with the heart rate of rats in LPS group at 4 h (*P*>0.05). AniHBr significantly increased the mean atrial pressure (Control group, 116.7 ± 9.2 mmHg; LPS group, 86.8 ± 4.0 mmHg; LPS+AniHBr, 118.9 ± 12.7 mmHg; Figure 2C) and reduced the heart rate (Control group, 418.7 ± 7.1 s^−1^; LPS group, 508.3 ± 16.2 s^−1^; LPS+AniHBr, 412.8 ± 27.3 s^−1^; Figure 2D) at 24 h, compared with the LPS group. Overall, AniHBr improved hemodynamics in sepsis rats.

### AniHBr inhibits inflammation in sepsis rats

**Figure 3:**
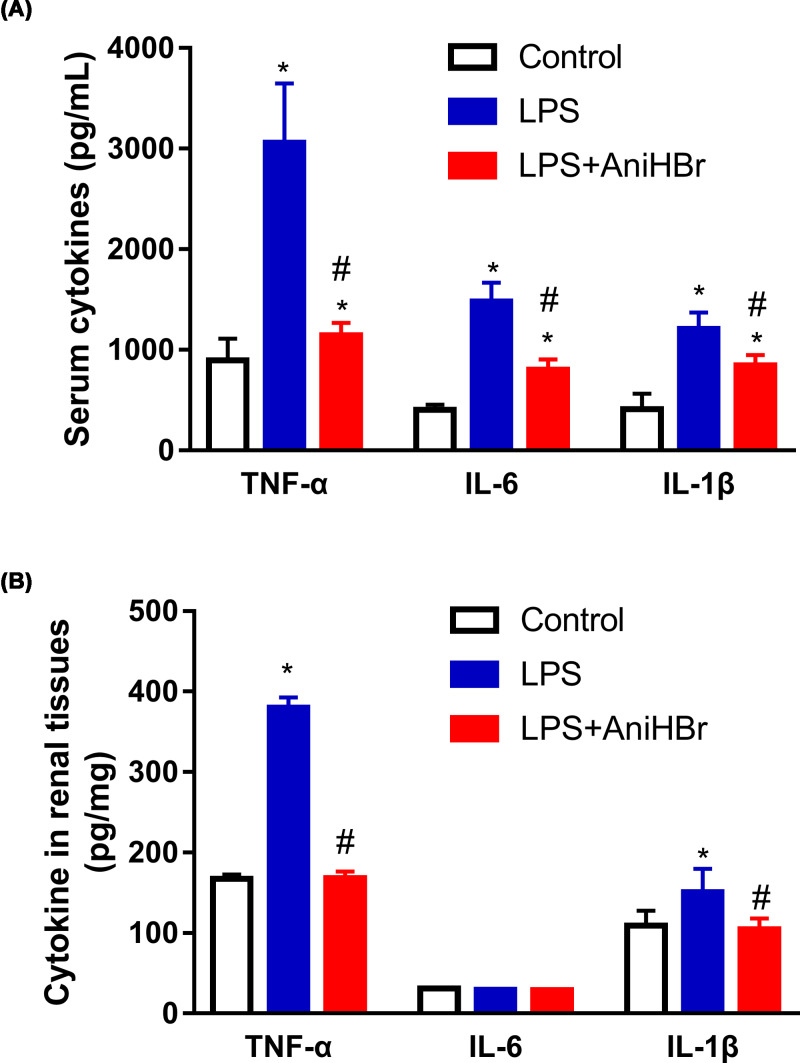
Serum and renal TNF-α, IL-1β and IL-6 levels at 24 h The rats were treated with LPS, and then administrated with vechicle or AniHBr. (**A**) The cytokine in serum. (**B**) The renal cytokine concentration is presented in mg of tissue wet. Mean ± SD, *P<0.05 vs. control; ^#^*P*<0.05 vs. LPS.

Inflammatory cytokines including TNF-α, IL-6 and IL-1β in serum and renal tissues specimens isolated from rats after LPS and AniHBr administration were detected (Figure 3). Compared with control rats, TNF-α, IL-6 and IL-1β in serum were significantly increased in the rats after LPS administration with or without AniHBr (Figure 3A). Notably, AniHBr administration significantly attenuated the LPS-induced secretion of serum TNF-α, IL-6 and IL-1β (Figure 3A). In renal tissues, TNF-α and IL-1β were demonstrated to significantly produce after the administration of LPS compared with the control group (Figure 3B). AniHBr adminstration significantly abolished the LPS-induced increases of TNF-α and IL-1β in renal tissues (Figure 3B). However, it was not observed significant differences in IL-6 levels among the control group, LPS group and LPS+AniHBr group.

### AniHBr alleviates LPS-induced renal damage

**Figure 4:**
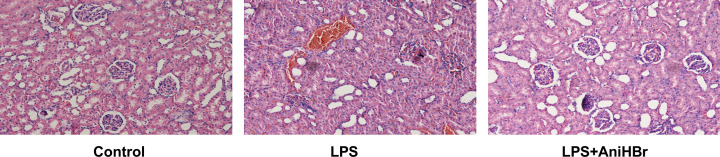
Effect of AniHBr in LPS-induced renal injury The rats were treated with LPS, and then administrated with vechicle, or AniHBr. Renal histology was performed after AniHBr administration**.**

Renal histology was performed after AniHBr administration (Figure 4). There were no pathological changes in the control group. Aggravated cortical tubular damages including tubular dilation, tubular necrosis, brush border loss, tubule degeneration and tubular cast formation were observed in kidney cortex and medulla after 24 h of LPS administration (Grade III). With AniHBr treatment, those histopathological changes were ameliorated (Grade I).

### AniHBr reduces MDA levels and increased SOD activity after LPS-induced renal injury

**Figure 5:**
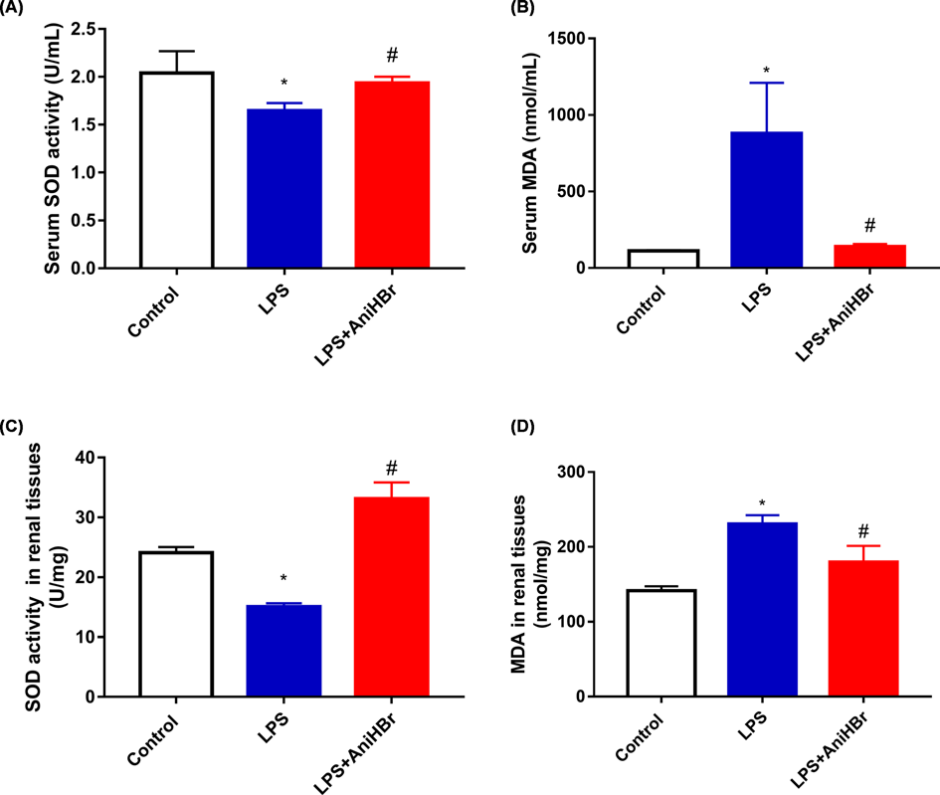
Serum and renal SOD, and MDA levels at 24 h The rats were treated with LPS, and then administrated with vechicle, or AniHBr. (**A**) Serum SOD activity. (**B**) Serum MDA. (**C**) SOD activity in renal tissues. (**D**) MDA in renal tissues. Mean ± SD, *P<0.05 vs. control; ^#^*P*<0.05 vs. LPS.

The antioxidative effect of AniHBr in LPS rats was investigated using ELISA (Figure 5). Compared with the control group, in both serum and renal tissues, the SOD activity was significantly reduced (Figure 5A,C) and MDA was significantly increased (Figure 5B,D) in LPS group. However, AniHBr administration present an opposite effect on SOD and MDA in both serum and renal tissues. The serum and renal tissues levels of SOD and MDA were significantly reversed by AniHBr administration in the LPS rats.

### AniHBr alleviated TNF-α inhibited oxidative phosphorylation

**Figure 6:**
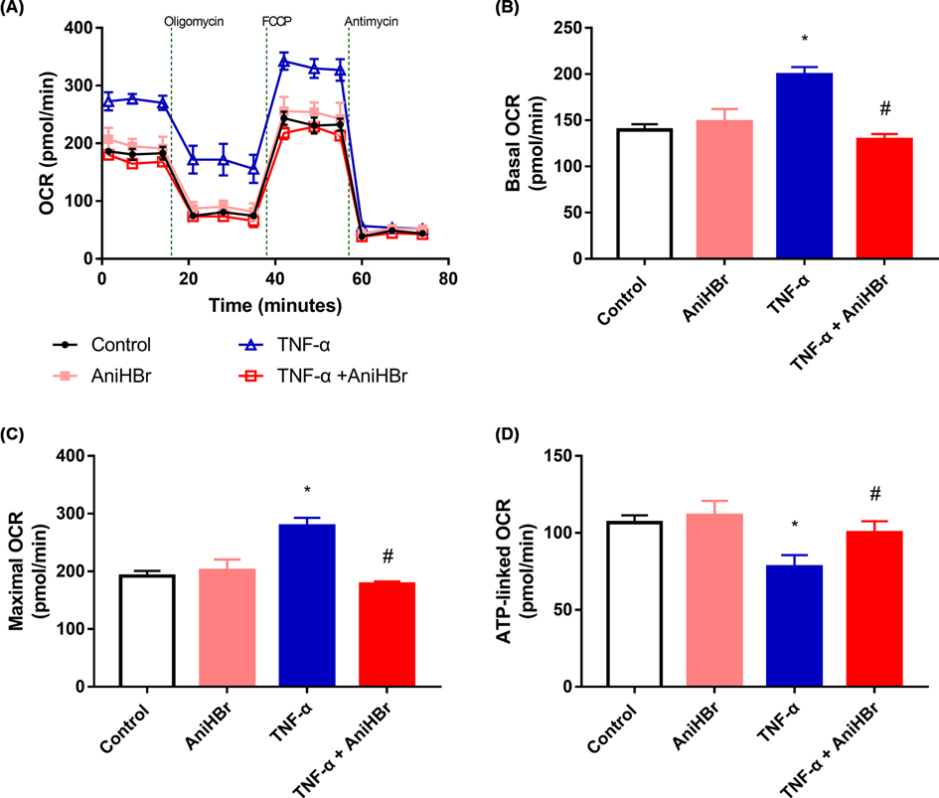
AniHBr rescued TNF-α inhibited metabolic switch Cells were treated with or without a final concentration of 10 μg/ml AniHBr in the presence of a final concentration of 10 ng/ml TNF-α for 24 h. Then, the mito stress test was performed. (**A**) Oxygen consumption rate (OCR) curves. (**B**) Basal OCR. (**C**) Maximal OCR. (**D**) ATP-linked OCR. *n*=3, Mean ± SD, *P<0.05 vs. control; ^#^*P*<0.05 vs. TNF-α.

Mito stress tests (Figure 6A) showed that the basal OCR (Figure 6B) and maximal OCR (Figure 6C) in the 10 ng/ml TNF-α induction group were significantly increased, and ATP-linked OCR (Figure 6D) was significantly reduced, compared with in control group. In the presence of AniHBr, basal OCR, maximal OCR and ATP-linked OCR were significantly reversed compared with the TNF-α induction group. The basal OCR, maximal OCR and ATP-linked OCR were not significantly changed in rats with the addition of AniHBr, compared with the control rats.

**Figure 7:**
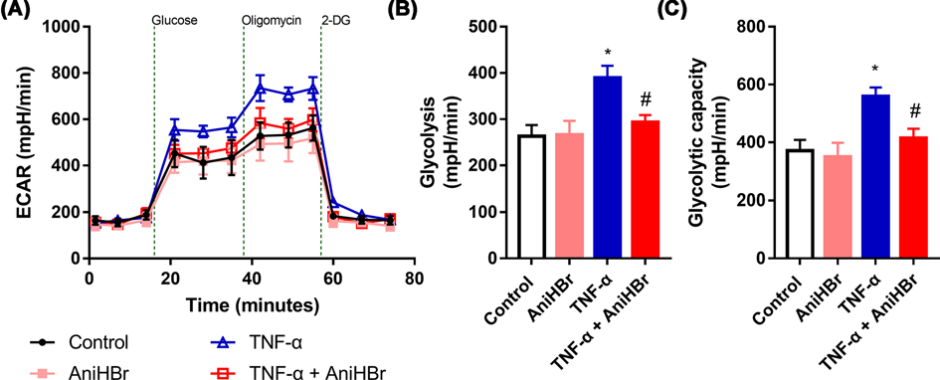
AniHBr rescued TNF-α inhibited glycolysis and glycolytic capacity Cells were treated with or without a final concentration of 10 μg/ml AniHBr in the presence of a final concentration of 10 ng/ml TNF-α for 24 h. Then, the glycolysis was measured and analyzed. (**A**) Extracellular acidification rate (ECAR). (**B**) Glycolysis. (**C**) Glycolytic capacity. *n*=3, Mean ± SD, *P<0.05 vs. control; ^#^*P*<0.05 vs. TNF-α.

Glycolysis stress tests (Figure 7A) showed that the glycolysis (Figure 7B) and glycolytic capacity (Figure 7C) in the presence of 10 ng/ml TNF-α was significantly increased, while it was not observed significant changes with the addition of AniHBr compared with the control group, suggesting the increase of glycolysis and glycolytic capacity by TNF-α was attenuated by the addition of AniHBr.

## Discussion

Tissue hypoxia and mitochondrial dysfunctions are thought to be important mediators of sepsis-induced multiple organ dysfunction [16]. In recent, it was contradicted renal ischemia contributed to AKI [17]. Renal blood flow is even increased in septic AKI, suggesting intrarenal vasodilation together with microcirculatory changes lead to renal functional changes [17]. Nevertheless, inflammation and mitochondrial dysfunction was closely associated with AKI [18,19]. Nowadays, there was no available specific pharmacological therapy for renal protection [17]. In the present study, we reported that AniHBr inhibits inflammatory cytokines and thus protects against AKI by attenuating mitochondrial dysfunction.

Anisodamine administration could improve the hemodynamics, maintain the histology by inhibiting the consecutive tubular necrosis and protect against functional impairment. Raised lactate concentration is an important prognostic marker for sepsis, and the reduction of lactate content is associated with improved outcomes [20]. AniHBr significantly suppressed the serum creatine kinase and lactate concentrations in rats with administration of intravenous LPS, showing a protective effect in LPS-induced rat septic AKI. The LD_50_ (median lethal dose) of AniHBr i.v. administration is 742 mg/kg. Thus, the multiply injection of AniHBr is safe.

During sepsis, inflammation mediators will not only activate the immune systems but also mediate host cellular injury. TNF-α elicited by LPS might be responsible for the septic shock [21]. Depletion of M2 macrophage could induce the secretion of TNF-α during sepsis-induced AKI, and significantly suppress the proliferation of tubular cells [22], suggesting TNF-α is associated with the tubular cell necrosis. It was demonstrated that mitochondrial dysfunction could decrease ATP generation, enhance mitochondrial oxidative stress, cell necrosis and apoptosis, and thus contributes to AKI [19]. Amelioration of mitochondrial dysfunction could inhibit the tubule epithelial cell apoptosis and protect LPS-induced AKI [18]. Oxidative phosphorylation takes place in the inner mitochondrial membrane where electron transport chain lies. The transfer of electrons leads to ATP production. Molecular oxygen is the final receptor of the electrons. Thus, oxygen consumption rate was used to assess the mitochondrial function [23]. It was demonstrated that proinflammatory TNF-α induced oxygen consumption *in vitro* HEK293 cells [24]. AniHBr inhibited the TNF-α induced oxygen consumption.

Mitochondria is a main source of intracellular ROS. In contrast-induced AKI, there was a protective effect against AKI by attenuating mitochondrial dysfunction and oxidative stress [25]. AniHBr significantly increased SOD activity and reduced the MDA concentration in the serum and renal tissues in septic rats. In the presence of TNF-α, oxygen consumption rate was significantly increased, and ATP-linked oxygen consumption rate (ATP production) was significantly reduced. In consistent to the change in mito stress, glycolysis and glycolysis capacity was significantly increased by TNF-α, suggesting proinflammatory cytokine TNF-α induced mitochondrial dysfunction, enhanced mitochondrial oxidative stress and glycolysis. AniHBr significantly inhibited those changes induced by TNF-α. Thus, AniHBr is an efficient anti-inflammatory and antioxidant agent. The molecular mechanism of AniHBr in protection of tubular cell necrosis should be investigated in the future. Moreover, the molecular mechanism by which AniHBr can significantly attenuate LPS-induced TNF-α, IL-6 and IL-1β in serum and attenuate oxidative stress will be investigated in the future.

The anti-inflammatory effect of anisodamine had been reported in LPS-induced pancreatic acinar cells [26]. Anisodamine might play a significant role in LPS-induced microvascular endothelial cells [27]. Endothelial cells interact with other cells via exosome [28]. Anisodamine acts as a cholinergic antagonist to improve smooth muscle function [29]. In recent, it was demonstrated that antagonist of M2 subtype muscarinic cholinergic receptors (AF-DX116) might accelerate LPS-induced lung and hepatic injuries, and antagonist of M1 subtype muscarinic cholinergic receptors (pirenzepine) and non-selective competitive antagonist atropine relieved those injuries [30]. The interplay of microvascular dysfunction and inflammation, and oxidative stress might lead to mitochondrial dysfunction via muscarinic cholinergic receptors. Future biomarkers for prevention and therapy of AKI may represent a better protection of the microvasculature and maintain the function of the endothelium.

## Conclusion

In the present study, we concluded that inflammation, mitochondrial dysfunction and oxidative stress are involved in LPS-induced AKI, and AniHBr protects against the LPS-induced inflammatory cytokines, mitochondrial dysfunction and oxidative stress, and thus attenuates the LPS-induced AKI. AniHBr might improve the balance in energy utilization via modulation of inflammation, which is a promising therapeutic drug for the treatment of LPS-induced acute kidney injury.

## Data Availability

All data generated or analyzed during this study are included in this published article.

## Abbreviations

2-DG: 2-deoxyglucose
AKI: acute kidney injury
AniHBr: anisodamine hydrobromide
ANOVA: analysis of variance
ATP: adenosine triphosphate
ECAR: extracellular acidification rate
FCCP: carbonylcyanide p-(trifluoromethoxy) phenylhydrazone
LPS: lipopolysaccharide
LSD: least significant difference
MDA: malondialdehyde
OCR: oxygen consumption rate
ROS: reactive oxygen species
SOD: superoxidedismutase
TNF-α: tumor necrosis factor α
